# Differential neuronal expression of receptor interacting protein 3 in rat retina: involvement in ischemic stress response

**DOI:** 10.1186/1471-2202-14-16

**Published:** 2013-02-02

**Authors:** Ju-Fang Huang, Lei Shang, Meng-Qi Zhang, Hui Wang, Dan Chen, Jian-Bin Tong, He Huang, Xiao-Xin Yan, Le-Ping Zeng, Kun Xiong

**Affiliations:** 1 Department of Anatomy and Neurobiology, School of Basic Medical Sciences, Central South University, Changsha, Hunan, 410013, China; 2 Eight-year Clinical Medicine Program, Class 2002, Central South University Xiangya School of Medicine, Changsha, Hunan, 410013, China; 3 Department of Histology and Embrology, School of Basic Medical Sciences, Central South University, Changsha, Hunan, 410013, China

**Keywords:** Receptor-interacting protein 3, Retina, Necroptosis, aHIOP

## Abstract

**Background:**

Receptor-interacting protein 3 (RIP3), a member of RIP family proteins, has been shown to participate in programmed necrosis or necroptosis in cell biology studies. Evidence suggests that necroptosis may be a mode of neuronal death in the retina.

**Results:**

In the present study we determined the expression of RIP3 in normal rat retina and its changes following acute high intraocular pressure (aHIOP). RIP3 immunoreactivity (IR) was largely present in the inner retinal layers, localized to subsets of cells expressing neuron-specific nuclear antigen (NeuN), parvalbumin and calbindin in the ganglion cell layer (GCL) and inner nuclear layer (INL). No double labeling was detected for RIP3 with PKC-α or rhodopsin. RIP3 immunoreactivity was increased in the GCL at 6 hr and 12 hr, but reduced at 24 hr in the retina, without apparent alteration in laminar or cellular distribution pattern. Western blot analysis confirmed the above time-dependent alteration in RIP3 protein expression. RIP3 expressing cells frequently co-localized with propidium iodide (PI). A few co-localized cells were observed between RIP3 and Bax or cleaved caspase-3 in the GCL in 12 hr following aHIOP.

**Conclusions:**

The results indicate that RIP3 is expressed differentially in retinal neurons in adult rats, including subsets of ganglion cells, amacrine and horizontal cells. RIP3 protein levels are elevated rapidly following aHIOP. RIP3 labeling co-localized with PI, Bax or cleaved caspase-3 among cells in the ganglion cell layer following aHIOP, which suggest its involvement of RIP3 in neuronal responses to acute ischemic insults.

## Background

Retinal neuronal damage and death are common to a variety of ophthalmological and neurological conditions, including retinal injury following acute high intraocular pressure (aHIOP, a classic ischemia model), retinal pigment epithelium detachment, vascular and age-related neuronal degenerations [1-3]. Necrosis can be found at the early stage of aHIOP. Necrosis has been considered an accidental and uncontrolled form of cell death for a long time. However, evidence also shows that necrotic cell death is sometimes controlled as caspase-dependent apoptosis, named it as necroptosis or programmed necrosis. By using the Necrostain-1 (a kind of inhibitor of necroptosis) and propidium iodide (PI)-staining after retina ischemia, Rosenbaum *et al.* have found that necroptosis is considered as an important mode of neuronal cell death during aHIOP, and autophagy deficit may be involved in this process [4].

Study of cellular necroptosis mechanism has been linked to RIP (Receptor-interacting protein) family and its relative molecular pathways. The receptor-interacting protein 3 (RIP3, also known as RIPK-3) is originally cloned from the human fetal brain and aortic endothelium and identified as a member of RIP family [5]. Subsequently, RIP3 is characterized as a N-terminal Serine/Threonine kinase capable of perceiving variations in internal cellular environment and participating in cell survival or death signaling [6]. RIP3 binds to and induces RIP1 phosphorylation, and thus activates the nuclear factor-kappa B (NF-κB) [5,7-9]. RIP3 may play a key role in necroptosis by activating tumor necrosis factor α (TNF-α) [10-12]. Other data suggest that RIP3 activation may perturb energy metabolism by up-regulating glycogen phosphorylase (PYGL) and glutamate dehydrogenase, which in turn potentiates superfluous α-ketoglutarate production and glucose phosphorylation, and eventually accelerates the Krebs cycle in mitochondria leading to excessive genesis of reactive oxygen species (ROS).

Base on the unknown molecular mechanisms of retinal neuronal necroptosis at the early stage of aHIOP, we wondered whether RIP3 alteration may be involved in cell death at the early stage of aHIOP. In the present study we first characterized the cellular localization of RIP3 in adult rat retina, and then detected the changes of RIP3 expression relative to cell death at early stage of ischemia following aHIOP.

## Methods

### Animals

Twenty-four adult Sprague–Dawley rats weighing 200–250 grams, available from the animal center of Central South University, were used in the present study. Rats were randomly divided into the control group (n=6) and experimental group (n=18) subjected to induction of acute high intraocular pressure (aHIOP). All animals were housed in acrylic box cages with free access to food and water. Animals were maintained under conditions of constant temperature (25°C), humidity (50±10%) and lighting cycle (12:12 hours). All experimental procedures used in the present study were approved by Ethics Committee of Xiangya School of Medicine, in accordance with the NIH guidelines for use and care of laboratory animals.

### Induction of acute high intraocular pressure (aHIOP) and propidium iodide treatment

The animal model was prepared following the procedure described by Tong [13]. In brief, Animals were anesthetized with 10% chloral hydrate (0.2 ml/kg). A drop of chloramphenicol was administered to the conjunctive sac. A 30-gauge needle connected to the instillation instrument filled with normal saline was inserted into the anterior chamber. The intraocular pressure (IOP) was elevated to 110 mmHg, maintained for 60 min, and then gradually lowered to normal. The rats were allowed to survive for 6, 12 and 24 hrs before terminal use. Thirty minutes prior to animal perfusion, 5 μl PI (1.0 mg/ml in DW, Sigma, MO, USA) was administered by intravitreal injection.

### Tissue preparation

For anatomical examination, animals were deeply anesthetized with 10% Chloral hydrate (Sinopharm Chemical Reagent Co. Ltd, Shanghai, China) in saline (0.4 ml/kg, i.p.), followed by trans-cardiac perfusion with saline and then 4% paraformaldehyde in 0.1 M phosphate buffer (PB, pH 7.4) at different survival time points. The eyeballs were enucleated, and the cornea, lens and vitreous body were removed. The remaining eye cups were postfixed in 4% PF overnight, and immersed in ascending sucrose solutions (15% to 30%) in 0.1 M PB at 4°C for cryoprotection. The eye cups were embedded in Optimal-Cutting-Temperature (OCT) medium (Sakura Finetek, Japan), prepared into 20 μm thick cross-sections in a Shanton cryostat (Thermo-Fisher Scientific Inc., CA, USA). Sections were thaw-mounted on positively charged microslides (Thermo-Fisher Scientific Inc., CA, USA), allowed to air-dry and then stored at −20°C before further histological processing. For western blot analysis, eyeballs were removed immediately from deeply anesthetized rats after a vascular rinse with cold saline, and the retinas were dissected out and snap-frozen on dry-ice and stored at −70°C until tissue homogenization.

### Immunohistochemistry

For immunolabeling with the avidin-biotin complex (ABC) method, sections were treated in 0.3% H_2_O_2_ in 0.01M phosphate buffered saline (PBS, pH 7.3) for 15 minute to inactivate endogenous peroxidase. Non-specific antibody binding was blocked by a pre-incubation of sections in 5% normal horse serum (Sigma, MO, USA) in PBS containing 0.3% Triton X-100 (Fluka, CA, USA) for 1 hour at room temperature. Sections were then incubated with a rabbit anti-RIP3 antibody at 4°C overnight (Table 1), then reacted with biotinylated horse anti-rabbit IgG (1:400, Vector Laboratories Inc., CA, USA) for 2 hours at room temperature. After 1 hour incubation with the avidin-biotin complex reagents (1:400, Vector Laboratories Inc., CA, USA), immunoreaction product was visualized in PBS containing 0.05% DAB (Sigma, MO, USA) and 0.03% H_2_O_2_. Finally, the sections were dehydrated, cleared and coverslippered. The specificity of the RIP3 antibody was evaluated by pre-absorption and omission of the primary antibody in immunohistochemistry. Western blot was also used to confirm the antibody binding product.

**Table 1:** Primary antibodies used in the present study

**Antibody**	**Host**	**Source**	**Dilution**
Receptor-interacting protein 3 (RIP3)	Rabbit	Sigma-Alorich, PRS2283	(1:200)
Receptor-interacting protein 3 (RIP3)	Goat	Santa Cruz, sc-47364	(1:500)
Calbindin (CB)	Mouse	Sigma-Alorich, C9848	(1:4000)
CD11b	Mouse	Abcam, ab78457	(1:500)
Glial fibrillary acidic protein (GFAP)	Mouse	Abcam, ab10062	(1:1000)
Glutamine synthetase (GS)	Mouse	Abcam, ab64613	(1:5000)
Neuron-specific nuclear antigen (NeuN)	Mouse	Abcam, ab104224	(1:1000)
Protein kinase C alpha (PKCα)	Mouse	Abcam, ab86715	(1:500)
Parvalbumin (PV)	Mouse	Sigma-Alorich, P3088	(1:4000)
Rhodopsin(Rho)	Mouse	Sigma-Alorich, R5403	(1:1000)
Synaptophysin(Syn)	Mouse	Sigma-Alorich, S5768	(1:2000)
β-Tubulin	Rabbit	Abcam, ab6046	(1:1000)
Cleaved caspase-3	Rabbit	Millipore, AB3623	(1:200)
Bax	Mouse	Sigma-Alorich, B8429	(1:200)

For double immunofluorescence of RIP3 co-localization in normal retina, sections were pre-incubated for 60 minutes in 5% donkey serum (Sigma, MO, USA) in PBS containing 0.3% Triton X-100 at room temperature. Sections were incubated with the rabbit anti-RIP3 antibody and one of the mouse antibodies to neuronal and glial markers overnight (listed in Table 1). After several times rinses with PBS, sections were reacted with Cy2 and Cy3-conjugated donkey anti-mouse and anti-rabbit secondary antibodies at 1:200 (Invitrogen, CA, USA). The sections were then briefly incubated in Bisbenzimide (Hoechst 33258, Sigma, MO, USA) at 1:50,000, washed in PBS and covered with an anti-fading mounting medium (Abcam, MA, USA) before microscopic examination.

For immunofluorescence of RIP3 and double labeling of RIP3 with Bax or cleaved caspase-3 following aHIOP, sections were pre-incubated for 60 minutes in 5% donkey serum (Sigma, MO, USA) in PBS containing 0.3% Triton X-100 at room temperature. Sections were incubated with the rabbit anti-RIP3 antibody (for RIP3 expression test) or goat anti-RIP3 and Bax or cleaved caspase-3 antibodies overnight. After several times rinses with PBS, sections were reacted with Cy2 and Cy3-conjugated donkey anti-rabbit/donkey anti-goat/donkey anti-mouse secondary antibodies at 1:200 (Invitrogen, CA, USA). The sections were then briefly incubated in Bisbenzimide, washed in PBS and covered with an anti-fading mounting medium (Abcam, MA, USA) before microscopic examination.

For double labeling of PI-labeled cells and RIP3 following aHIOP. 12 hr of PI-pretreated retinal sections were selected to carry out RIP3 immunofluorescence staining. The protocol of RIP3 immunofluorescence staining have illustrated before. Photograph were using a confocal microscope (Nikon, DIGITAL ECLIPSE C1 plus, Japan) fitted with excitation/emission filters 568/585 for PI.

### Immunoblotting

Retinas were homogenized by sonication on ice in a digestion buffer [150 mM NaCl, 25 mM Tris- HCl (pH 7.4), 2 mM EDTA, 1.0% Triton X-100, 1.0% sodium deoxycholate, 0.1% SDS] containing a cocktail of protease inhibitors (Sigma, MO, USA). Homogenates were centrifuged at 10,000×*g* for 20 min at 4°C. The supernatants were collected, and protein concentration determined by Bicinnchoninic acid (BCA) assay (Pierce, IL, USA). A total of 100 μg of protein in 62.5 mM Tris loading buffer (pH 6.8, containing 25% glycerol, 2% SDS, 0.01% bromophenol blue and 5% β-mercaptoethanol, Bio-Rad, CA, USA), was boiled for 10 min, and loaded in each lane of 4-20% linear gradient Tris–HCl ready gel (Bio-Rad, CA, USA). The polypeptides were electrotransferred to Trans-Blot® pure nitrocellulose membrane (Bio-Rad, CA, USA). Non-specific binding was blocked with PBS containing 5% nonfat milk (Bio-Rad, CA, USA) and 3% bovine serum albumin (Sigma, MO, USA). Membranes were incubated with RIP3 or β-tubulin antibodies overnight, and then in HRP-conjugated secondary antibodies (1:20000, Bio-Rad, CA, USA) for 1 hour. Immunoblotting products were visualized with an ECL Plus™ Western Blotting Detection kit according to manufacturer’s instruction (GE Healthcare Life Sci., NJ, USA), and images captured in a Molecular Dynamics Phosphor imager (Nucleo Tech Inc., CA, USA). For RIP3 antibody specific test, nitrocellulose membranes were blotted for RIP3 with the presence of the immunogenic peptide corresponding to amino acids 473 to 486 of murine RIP3 (US Biological, R2031-74P, at 1:10 dilution).

### Imaging and data analysis

Sections were examined on an Olympus (BX53, Tokyo, Japan) microscope equipped with a digital camera and imaging system (CellSens Standard, Olympus, Tokyo, Japan) for single (DAB) immunolabeling and on a confocal microscope (Nikon, DIGITAL ECLIPSE C1 plus, Japan) for RIP3 distribution double immunolabeling and RIP3 immunofluorescence following aHIOP. For double labeling of Bax or cleaved caspase-3 with RIP3, the slices were examined with a confocal microscope (Carl Zeiss, Axiovert 200M, Germany). In the latter case, immunofluorescence was scanned from 1 μm depth of tissue (i.e., 5–6 μm below the section surface) around the middle segment of retina between the optic nerve disk and periphery. Semi-quantitative analyses were conducted using approximately 20 merged images (40×) from 4 animals (5 sections per animal) to estimate the frequency of colocalization. Figure panels were assembled using Photoshop CS5 (Adobe Systems Incorporated, CA, USA). Image J (National Institutes of Health, MD, USA) were used to analyze the optical density value of the RIP3 bands by western blot. The average values of RIP3 and β-Tubulin were compared, the average relative value was obtained. One-way analysis of variance was performed to test differences between group averages. All results were presented as mean ± SD. A value of *p*<0.05 was considered statistically significant.

## Results

### Overall laminar distribution of RIP3 immunoreactivity in retina

**Figure 1:**
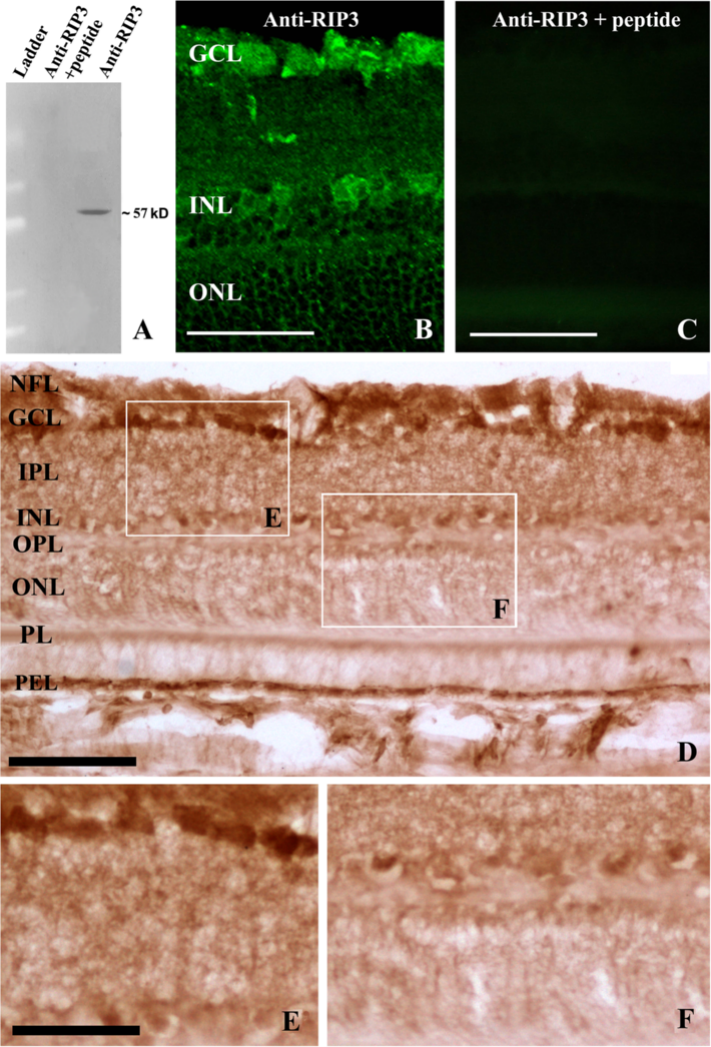
**Antibody characterization (A-C) and overall laminar distribution of RIP3 immunolabeling in the adult rat retina (D-F).** Panel (**A**) shows an immunoblotting test of the antibody, which reveals a single band migrated at 57 kDa, blockable by preincubation of the immunogenic peptide with primary antibody. Panels (**B** and **C**) show that the antibody labeling (**B**) can also be eliminated by the pre-absorption peptide in immunofluorescent preparation. Panels (**D**) and enlargements (**E**, **F**) show RIP3 immunolabeling revealed by the avidin-biotin-peroxidase method. Labeling appears mostly in the inner retinal layers and the pigment epithelium layer (PEL) (**D**). Many labeled somata are present in the ganglion cell layer and some also seen in the inner nuclear layer (INL). PL: photoreceptor layer; ONL: outer nuclear layer; OPL: outer plexiform layer; INL: inner nuclear layer; IPL: inner plexiform layer; NFL, nerve fiber layer. Scale bar=70 μm in **B**, **C** and 100 μm in D, 50 μm in **E**, **F**.

The RIP3 antibody detected a monoband migrated approximately at 57 kDa, consistent with the previous reports regarding the molecular weight of the protein [8]. In the presence of the immunogenic peptide, this immunoblotting band was eliminated (Figure 1A). Similarly, RIP3 immunoreactivity in retinal cross-section could be abolished by pre-absorption of the primary antibody with the neutralizing peptide (Figure 1B,C), or omission of the primary or secondary antibody (data not shown).

Immunohistochemistry using the peroxidase-DAB method visualized a fairly broad laminar distribution of RIP3 IR in the retina. Overall, a strong cellular labeling was notable in the ganglion cell layer (GCL), with some labeled somata also seen in the inner nuclear layer (INL) (Figure 1B,D). Diffuse and moderately intense neuropil-like labeling was present in the inner plexiform layer (IPL). Weak labeling was found over the outer plexiform layer (OPL) to photoreceptor layer (PL). The pigment epithelium cells exhibited fairly strong labeling (Figure 1D-F).

### Double labeling for RIP3 and neuronal markers in retina

**Figure 2:**
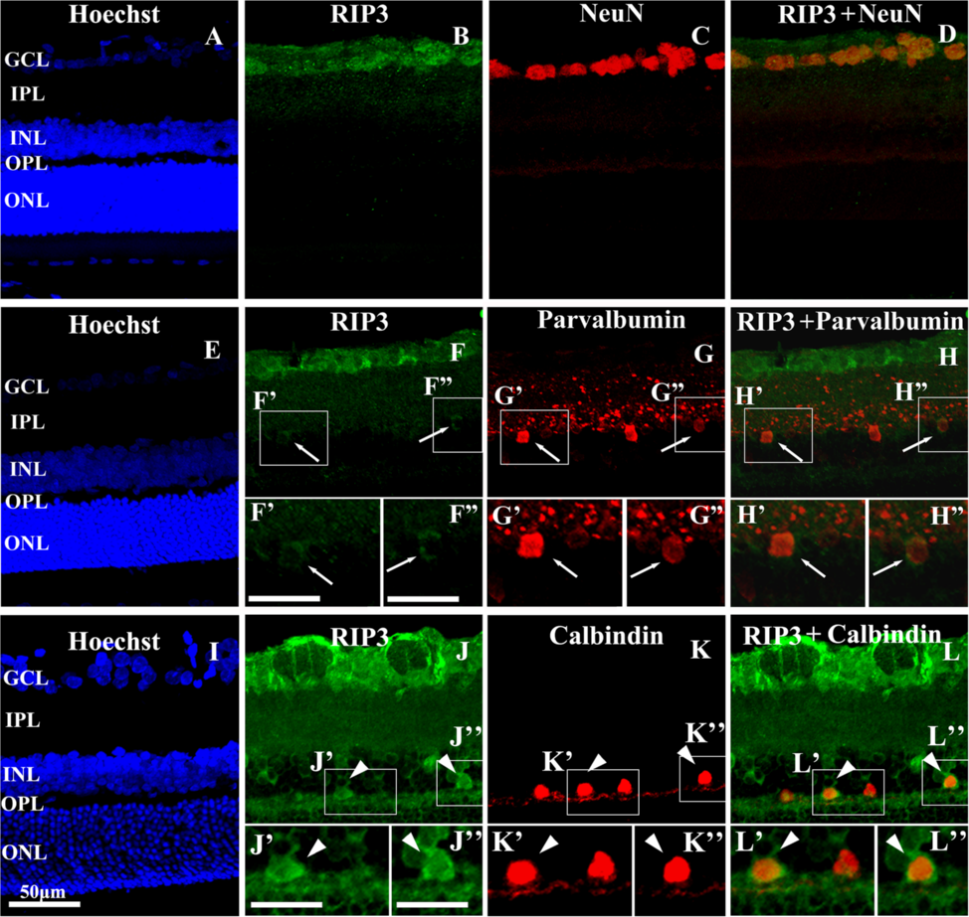
**Confocal double immunofluorescent images showing colocalization of RIP3 with NeuN (A-D), parvalbumin (E-H') and calbindin (I-L') in adult rat retina.** Hoechst nuclear labeling (blue) shows the laminas of the retina (**A**, **E**, **I**). Framed areas are enlarged as indicated. RIP3 immunoreactivity co-localize with NeuN frequently (**D**) in the ganglion cell layer, and partially with parvalbumin and calbindin in the inner nuclear layer. Scale bar=50 μm in **A**-**L**, Bar=25 μm in **F’**, **F”**; **G’**, **G”**; **H’**, **H”**. Abbreviations are as defined in Figure 1.

**Figure 3:**
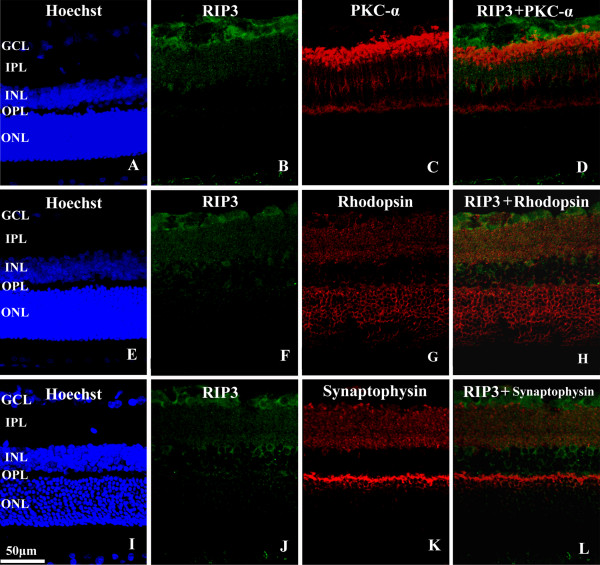
**Confocal images illustrating double labeling for RIP3 with protein kinase C-α (PKCα) (A-D), rhodopsin (E-H) and synaptophysin (I-L) in adult rat retina.** RIP3 labeling appears not to co-localize with PKCα in the inner nuclear layer. In the inner plexiform layer, light RIP3 immunofluorescence appears to be interposed with the strong immunoreactivity for PKCα (**D**), rhodopsin (**H**) and synaptophysin (**L**). Scale bar=50 μm in **A**-**L**. Abbreviations are as defined in Figure 1.

Confocal double immunofluorescence was carried out to determine RIP expression relative to markers of major neuron phenotypes in the retina. In the GCL (Figure 2A-D), the cellular labeling of RIP3 was found to be commonly co-localized with NeuN [14]. Preliminary cell count using high magnification (40X) images revealed that RIP3 labeling was found in approximately 42% of NeuN positive perikarya in the GCL (89/214). In the INL, RIP3 IR was found to coexist in amacrine-like or horizontal-like neurons that were immunoreactive either for parvalbumin (amacrine cell marker) (Figure 2E-H) or calbindin (horizontal cell marker) (Figure 2I-L)[15], counting for roughly 18% (23/126) and 41% (60/146) respectively. In contract, no co-localization of RIP3 IR was detected in the INL among neurons expressing PKCα (Figure 3A-D), a marker for bipolar cells [16]. Further, no distinct co-localization was found between RIP3 and rhodopsin in the photoreceptor cells [14] or their terminals (Figure 3E-H).

As seen with the peroxidise-DAB preparations (Figure 1D), weak RIP3 IR appeared in the inner and outer plexiform layers of the retina in confocal light microscopy (Figure 3B, F, J). In double immunofluorescence, this terminal-like RIP3 labelling was weaker relative to synaptophysin immunoreactivity in the IPL and OPL (Figure 3K). Close examination indicated that RIP3 and synaptophysin immunoreactivities appeared to be not co-localized in these two plexiform layers (Figure 3J-L).

### Double labeling for RIP3 and glial markers in retina

**Figure 4:**
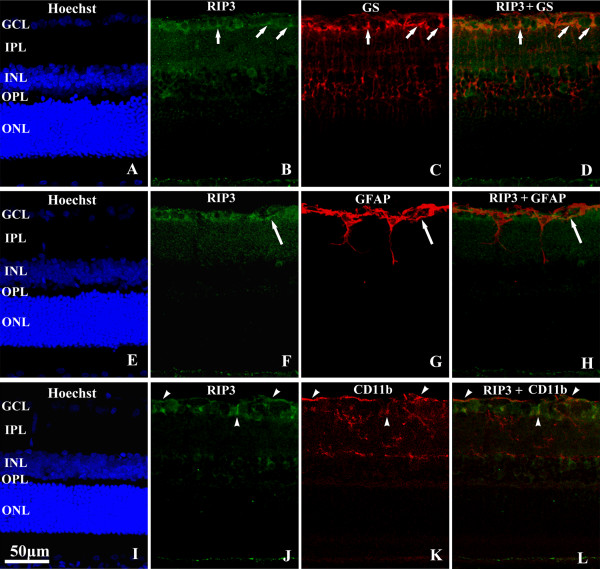
**Confocal light microscopic images illustrating RIP3 immunolabeling relative to immunolabeling of glial cells in adult rat retina (PKCα).** In the ganglion cell layer, RIP3 expressing somata appear to be tightly apposed to (or potentially co-localized with immunoreactivity for glutamine synthetase (**GS**) that visualizes Müller cells (**B**-**D**). A similar pattern is seen between RIP3 and GFAP (**F**-**H**) or RIP3 and CD11b (**J**-**L**) double immunofluorescence, these two glial markers are expressed in astrocyte and microglia in the retina. Scale bar=50 μm in **A**-**L**. Abbreviations are as defined in Figure 1.

In order to further determine whether RIP3 expression occurred in glial cells in retina, double immunofluorescence was performed for RIP3 with glutamine synthetase (GS), GFAP, CD11b, markers for Müller cells, astrocytes and microglia, respectively. RIP3 and GS immunolabeled elements apposed closely to each other in the GCL (Figure 4A-D), representing an interposing arrangement of the ganglion cells and Müller glia [17]. The might exist infrequent co-localization between the two markers (Figure 4B-D). GFAP immunoreactive astrocytes were distributed in nerve fiber layer and ganglion cell layer. No clear somata co-localization was detectable between RIP3 and GFAP labeling, although small process-like element appears to exhibit double labeling (Figure 4E-H). CD11b immunoreactivities were also largely located to the inner retina. Double labeling was detectable around the RIP3 immunoreactive somata or at process-like elements in the GCL and NFL (Figure 4I-L).

### Changes of RIP3 expression following aHIOP

**Figure 5:**
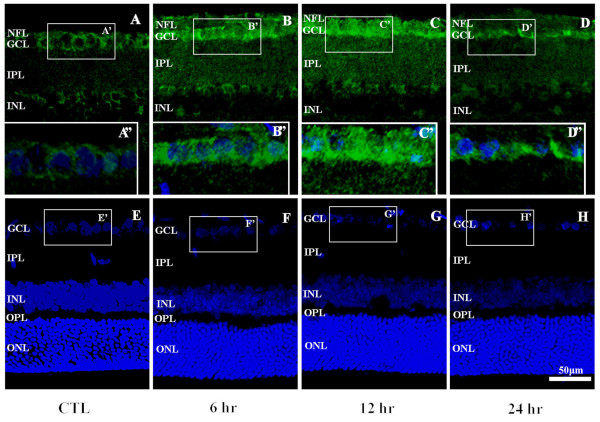
**Confocal microscopic images illustrating RIP3 expression following aHIOP.** The pattern of RIP3 expressing was not changed following aHIOP at different survival time point. In the ganglion cell layer, intensity of RIP3 immunoreactivity were appeared to be up-regulate in somata in 6 hr and 12 hr groups (**B**-**D**). Scale bar=50 μm in **A**-**H**, Scale bar=25 μm in **A**-**D**. Abbreviations are the same as detailed in Figure 1.

The immunofluorescence staining result showed that RIP3 was mainly present in ganglion cell layer (GCL), nerve fiber layer (NFL), inner plexiform layer (IPL) and inner nuclear layer(INL), but weakly staining occurs in other layers in retina (Figure 5), meanwhile, no difference in distribution was detected. Generally, in contrast with the normal controls, significantly more distinct and heavier RIP3 immunoreactive showed in aHIOP animal models, a strong cellular labeling of RIP3 in 6 hour and 12 hour groups was found, but weakly found in 24 hour groups.

**Figure 6:**
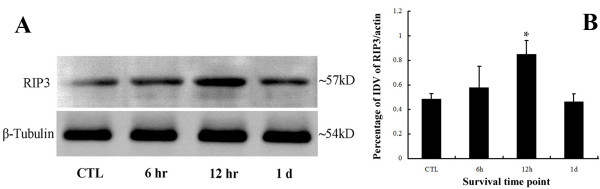
**Western blot analysis result of RIP3 following aHIOP.** The protein level of RIP3 is increased at 6 hr or 12 hr, and tends to be normal at 24 hr. Panel **A**: bands of RIP3 and β-tubulin; Panel **B**: optical density analysis of RIP3; Error bars represent standard deviation, 12hr *vs* other groups *p<0.05.

The western blot results showed that RIP3 was mainly exhibited as a single 57 kDa band in all groups (Figure 6A). The bands in aHIOP groups were apparently thicker and larger than those of normal control groups. The band in 24 hour groups was thinner and smaller than those in injury groups and tended to be normal. β-tubulin was mainly exhibited as a single 54 kDa band respectively in all four groups.

The optical density measurement and statistical analysis indicated that aHIOP up-regulated the expression of RIP3 in the early stage (Figure 6B). The values of the bands density showed as following: Normal control (0.52±0.13), 6 hr (0.59±0.24), 12 hr (0.81±0.18) and 24 hr (0.41±0.14). It demonstrated that RIP3 bands density were increased firstly and then decreased as time extend within 1day.

### Double labeling for RIP3 and PI in retina following aHIOP at 12 hr

**Figure 7:**
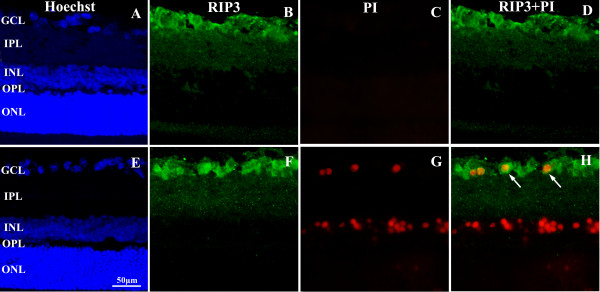
**Confocal microscopic images illustrating double labeling of RIP3 and propidium iodide (PI) positive cells following aHIOP at 12 hr.** The pattern of RIP3 expression appears comparable relative to normal controls. In the ganglion cell layer, intensity of RIP3 immunoreactivity appears somewhat up-regulated in neuronal somata, and commonly colocalizes with PI positivity (**H**). Scale bar=50 μm for panel **A**-**H**. Abbreviations are the same as detailed in Figure 1.

PI-positive cells were largely detected in the ganglion cell layer (GCL) and inner nuclear layer (INL) in 12 hr group (Figure 7). PI-stainings were localized in nuclei, as confirmed with bisbenzimide stain, suggestive of the presence of necrosis cells in GCL and INL after 12 hr post-ischemia. RIP3 and PI co-localization was found in GCL, with some PI-positive cells exhibited very strong RIP3 (white arrow show).

### Double labeling for RIP3 and Bax or cleaved caspase-3 in retina following aHIOP at 12 hr

**Figure 8:**
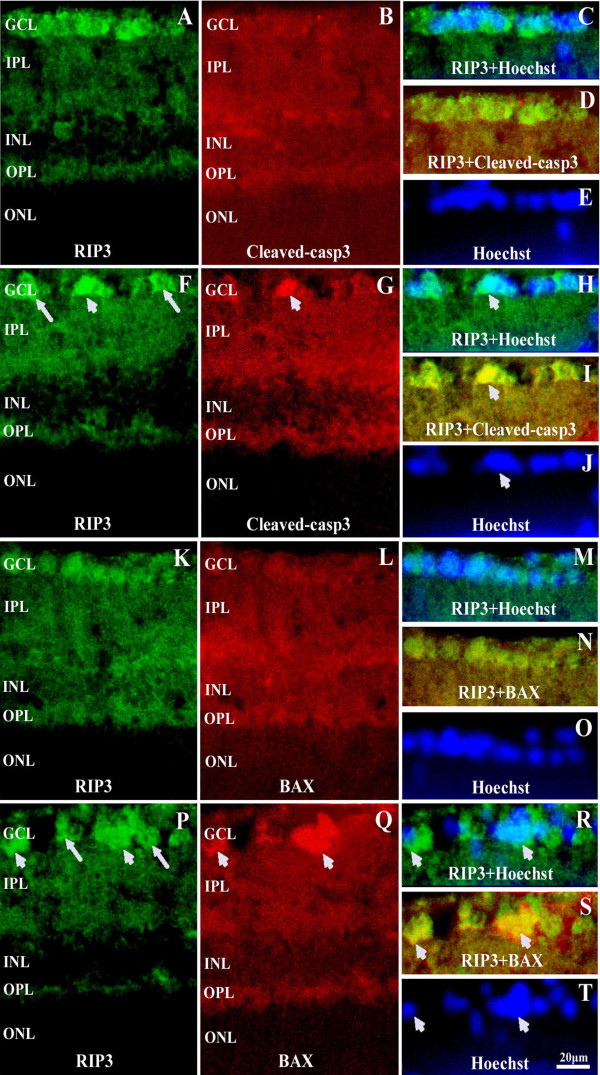
**Confocal microscopic images illustrating double labeling of RIP3 with Bax or cleaved caspase-3 positive cells following aHIOP at 12 hr.** Compared to control group (Panel **A**-**E**, Panel **K**-**O**), Bax (Panel **P**-**T**) and cleaved caspase-3 (Panel **F**-**J**) positive cells occur in GCL in 12 hr group. Some Bax or cleaved caspase-3 positive cells show strong RIP3 (arrow head shown). Some strong positive staining of RIP3 did not presence Bax or cleaved caspase-3 strong expression (arrow shown). Scale bar=20 μm in all panels. Abbreviations are as defined in Figure 1.

There was no distinct cellular staining of Bax and cleaved caspase-3 in normal rat retina (see Figure 8). Bax and cleaved caspase-3 positive cells were detected in GCL in 12 hr group, both of them were localized to nuclei, as confirmed with bisbenzimide staining. RIP3 labeling was found to colocalization with Bax or cleaved caspase-3 in GCL. Some Bax or cleaved caspase-3 positive cells exhibited strong RIP3 (arrow head shown). However, some strongly labeled RIP3 cells did not exhibit Bax or cleaved caspase-3 reactivity (arrow shown).

## Discussions

The retina is a part of the central nerve system and contains many types of neurons and glia cells with their somata and processes arranged in a stratified manner [18,19]. The retina has been used as an *in vivo* model for studying neuron stress and/or death in the central nervous system [13,20-23]. Also, retinal neuronal degeneration itself is characterized in many clinical conditions that can lead to blindness [24]. Understanding the molecular mechanism underlying cell stress and death in the retina may help develop therapeutic intervention for visual impairment. Neuronal death owing to necrosis and apoptosis may exist in the retina under pathological conditions [25]. For instance, high intraocular pressure may cause necrosis and delayed apoptosis in the retina, as characterized in experimental models [26-28]. More recently, it is suggested that programmed necrosis, also called necroptosis, may occur at the early stage of aHIOP [4]. This lately-recognized type of cell death may play an important role in neuronal degeneration in the inner retinal layers, namely the ganglion cell and inner nuclear layers.

### RIP3 is mainly expressed in neurons in the inner layer of normal rat retina

This present study reveals that RIP3 is constitutively expressed in the adult rodent retina. The RIP3 IR is detected across most neuronal somata and terminal layers of the retina, with labeling presented largely in the neuronal components. There exists a differential RIP3 among retina neurons in normal adult rat retina. Thus, RIP3 IR is fairly abundant in the NFL and GCL, wherein the labeling is found largely in the ganglion cells. Some discretely labeled elements appear to be the glial cells in this inner retinal location, including Müller cells, astrocytes and microglia, although it is not certain whether the “co-localized profiles” are due to an proximity of the neuron-glia spatial arrangement, *e.g.*, the ganglion cells *vs* Müller cells in the GCL. The double labeling data in the present study also suggests a residential expression of RIP3 in subpopulations of neurons that contain parvalbumin or calbindin. On the contrary, bipolar cells generally do not appear to exhibit impressive RIP3 labeling. Moreover, photoreceptors appeared to express RIP3 at minimal levels under normal condition. The weak RIP3 reactivity in plexiform layers (more noticeable in the IPL) might relate to a dendritic labeling because there is no apparent co-localization of RIP3 and synaptophysin. Overall, the laminar and cellular RIP3 expression patterns appear to be compatible with a notion that the up-regulation of this molecule could occur largely in the inner retinal layers, and may mediate necroptosis there under certain conditions. It should be noted that RIP3 expression in the photoreceptor cells is low under normal condition, although a strong RIP3 expression exists in the retinal pigment epithelial cells. The relevance of this distribution pattern at the outer layer of retina to the reported up-regulation of RIP3 following pigment epithelium detachment remains an interesting issue to investigate [29]. Finally, we should mention that the distribution of RIP3 in normal adult retina was conducted by immunohistochemistry, which has limitations. Our future studies, such as *in situ* hybridization, are warranted to further understanding of RIP3 expression in the retina.

### RIP3 expression is up-regulated in retina ganglion cells at early stage of aHIOP

Data are considerably limited to date with regard to the expression of RIP3 in the brain under normal and pathological conditions [7,29]. Nonetheless, recent evidence suggests that RIP3 may serve an important cellular factor regulating cellular/neuronal stress and death, possible by mediating necroptosis. A recent study in the retina demonstrates an up-regulation of RIP3, up to 10 fold, in an experimental model of retinal detachment, suggestive of a potential implication for necroptosis [29]. Interestingly, in a preliminary gene-chip analysis we noticed an early but marked increase in RIP3 mRNA expression following aHIOP in rat retina (own unpublished data). In the present study, RIP3 protein levels begin to increase at 6 hr, and reached maximum at 12 hr (12 hr *vs* other groups, *p*<0.05), implicating an involvement of RIP3 modulation at the early stage of aHIOP. However, currently there is no suitable morphological marker for necroptosis *in vivo*[30]. Using PI staining that labels necrotic cells, the present study show that RIP3 expressing cells may also PI-positive in GCL at 12 hr following aHIOP. Our double labeling results show there is small extent of co-localization of RIP3 with Bax or cleaved caspase-3 in GCL. However, some RIP3 strong expression cells are not exhibit Bax or cleaved caspase-3 reactivities. This finding suggests that RIP3 regulation may be potentially involved in the process of neuronal injury or death. Previous studies have reported that over-expression of RIP3 may induce apoptosis [5,8,12], our results are consistent with these finding. However, our results show that not all RIP3-upregulated cells exhibit co-localization with apoptosis relative proteins (Bax or cleaved caspase-3), which indicated that some RIP3 over-expression may not induce apoptosis. Based on the evidence of co-localization between RIP3 strong expression cells and PI-staining (Figure 7), we speculate that RIP3 up-regulation may participate in RGCs necroptosis following aHIOP. However, because there is no specific marker for necroptosis, future studies are required to determine the precise role of early RIP3-upregulation in retinal injury and diseases [28,31,32].

## Conclusion

This present study demonstrates that RIP3 is expressed differentially in retinal neurons in adult rats, including subsets of ganglion cells, amacrine and horizontal cells. RIP3 expression in RGCs is up-regulated following aHIOP, suggesting an involvement of RIP3 in neuronal responses to acute ischemic insults.

## Competing interests

The authors declare that they have no competing interests.

## Authors’ contributions

LS, KX and JFH designed the experiment, LS and KX performed the experiment, JB T and LPZ performed the preliminary gene-chip analysis, HW and DC analyzed the data, LS and KX drafted the manuscript, HH and MQZ revised the manuscript and participated in paper modification, XXY revised the manuscript for English writing, all authors participated in critical revision of the manuscript and approved the final manuscript.

## Ackowledgements

This work was supported by the National Natural Science Foundation of China (No.81070729, No.81100663, No. 81141068) and Doctoral Foundation of Ministry of Education of China (No.20100162110067).
